# Supplementary material for: The Adequacy of Current Legionnaires’ Disease Diagnostic Practices in Capturing the Epidemiology of Clinically Relevant Legionella: A Scoping Review
Source: Pathogens. 2024 Oct 1;13(10):857. doi: 10.3390/pathogens13100857 (PMC11510479; doi:10.3390/pathogens13100857)
Supplement: Supplementary file 1 [file pathogens-13-00857-s001.zip › pathogens-3190619-supplementary.pdf]

Table of Contents

Supplementary Materials. 1

Table S1. Search history, databases, and search terms. Boolean operators are bolded, and keywords are listed in brackets. 1

Supplementary Materials S1. Review protocol 4

Supplementary Materials.

Table S1. Search history, databases, and search terms. Boolean operators are bolded, and keywords are listed in brackets.

Date	Search Terms	Engine
December 21, 2022	((incidence) **OR** (epidemiology) **OR** (prevalence) **OR** (frequency)) **AND** ((diagnosis) **OR** (detection)) **AND** (legionella) **AND** ((pneumonia) **OR** (lung infection) **OR** (community acquired pneumonia) **OR** (coinfection) **OR** (lung abnormality) **OR** (lung abnormality) **OR** (lung abnormalities)) **AND** ((immunocompromised) **OR** (immunosuppressed) **OR** (HIV) **OR** (human immunodeficiency virus) **OR** (AIDS) **OR** (Acquired immunodeficiency syndrome) **OR** (cancer) **OR** (type 2 diabetes) **OR** (transplant) **OR** (autoimmunity) **OR** (arthritis) **OR** (IBD) **OR** (inflammatory bowel disease))	PubMed
December 21, 2022	((incidence) **OR** (epidemiology) **OR** (prevalence) **OR** (frequency)) **AND** ((diagnosis) **OR** (detection)) **AND** (legionella) **AND** ((pneumonia) **OR** (lung infection) **OR** (community acquired pneumonia) **OR** (coinfection) **OR** (lung abnormality) **OR** (lung abnormality) **OR** (lung abnormalities))	PubMed
December 21, 2022	((incidence) **OR** (epidemiology) **OR** (prevalence) **OR** (frequency)) **AND** ((diagnosis) **OR** (detection)) **AND** (legionella) **AND** ((pneumonia) **OR** (lung infection) **OR** (community acquired pneumonia) **OR** (coinfection) **OR** (lung abnormality) **OR** (lung abn**or**mality) **OR** (lung abnormalities)) **AND** ((immunocompromised) **OR** (immunosuppressed) **OR** (HIV) **OR** (human immunodeficiency virus) **OR** (AIDS) **OR** (Acquired immunodeficiency syndrome) **OR** (cancer) **OR** (type 2 diabetes) **OR** (transplant) **OR** (autoimmunity) **OR** (arthritis) **OR** (IBD) **OR** (inflammatory bowel disease))	Cochrane Register of Controlled Trials

December 21, 2022	((incidence) **OR** (epidemiology) **OR** (prevalence) **OR** (frequency)) **AND** ((diagnosis) **OR** (detection)) **AND** (legionella) **AND** ((pneumonia) **OR** (lung infection) **OR** (community acquired pneumonia) **OR** (coinfection) **OR** (lung abnormality) **OR** (lung abnormality) **OR** (lung abnormalities))	Cochrane Register of Controlled Trials
December 22, 2022	Legionnaires' disease	ClinicalTrials.gov
December 22, 2022	(("epidemiology"[Subheading] **OR** "epidemiology"[All Fields] **OR** "incidence"[All Fields] **OR** "incidence"[MeSH Terms]) **OR** ("epidemiology"[Subheading] **OR** "epidemiology"[All Fields] **OR** "epidemiology"[MeSH Terms]) **OR** ("epidemiology"[Subheading] **OR** "epidemiology"[All Fields] **OR** "prevalence"[All Fields] **OR** "prevalence"[MeSH Terms]) **OR** ("epidemiology"[Subheading] **OR** "epidemiology"[All Fields] **OR** "frequency"[All Fields] **OR** "epidemiology"[MeSH Terms] **OR** "frequency"[All Fields])) **AND** (("diagnosis"[Subheading] **OR** "diagnosis"[All Fields] **OR** "diagnosis"[MeSH Terms]) **OR** detection[All Fields]) **AND** ("legionella"[MeSH Terms] **OR** "legionella"[All Fields]) **AND** (("pneumonia"[MeSH Terms] **OR** "pneumonia"[All Fields]) **OR** (("lung"[MeSH Terms] **OR** "lung"[All Fields]) **AND** ("infections"[MeSH Terms] **OR** "infections"[All Fields] **OR** "infection"[All Fields])) **OR** (("residence characteristics"[MeSH Terms] **OR** ("residence"[All Fields] **AND** "characteristics"[All Fields]) **OR** "residence characteristics"[All Fields] **OR** "community"[All Fields]) **AND** acquired[All Fields] **AND** ("pneumonia"[MeSH Terms] **OR** "pneumonia"[All Fields])) **OR** ("coinfection"[MeSH Terms] **OR** "coinfection"[All Fields]) **OR** (("lung"[MeSH Terms] **OR** "lung"[All Fields]) **AND** abnormality[All Fields]) **OR** (("lung"[MeSH Terms] **OR** "lung"[All Fields]) **AND** abnormality[All Fields]) **OR** (("lung"[MeSH Terms] **OR** "lung"[All Fields]) **AND** ("abnormalities"[Subheading] **OR** "abnormalities"[All Fields] **OR** "congenital abnormalities"[MeSH Terms] **OR** ("congenital"[All Fields] **AND** "abnormalities"[All Fields]) **OR** "congenital abnormalities"[All Fields]))) **AND** (("immunocompromised host"[MeSH Terms] **OR** ("immunocompromised"[All Fields] **AND** "host"[All Fields]) **OR** "immunocompromised host"[All Fields] **OR** "immunocompromised"[All Fields]) **OR** ("immunocompromised host"[MeSH Terms] **OR** ("immunocompromised"[All Fields] **AND** "host"[All Fields]) **OR** "immunocompromised host"[All Fields] **OR**	PubMed Central

	"immunosuppressed"[All Fields]) **OR** ("hiv"[MeSH Terms] **OR** "hiv"[All Fields]) **OR** ("hiv"[MeSH Terms] **OR** "hiv"[All Fields] **OR** ("human"[All Fields] **AND** "immunodeficiency"[All Fields] **AND** "virus"[All Fields]) **OR** "human immunodeficiency virus"[All Fields]) **OR** ("acquired immunodeficiency syndrome"[MeSH Terms] **OR** ("acquired"[All Fields] **AND** "immunodeficiency"[All Fields] **AND** "syndrome"[All Fields]) **OR** "acquired immunodeficiency syndrome"[All Fields] **OR** "aids"[All Fields]) **OR** ("acquired immunodeficiency syndrome"[MeSH Terms] **OR** ("acquired"[All Fields] **AND** "immunodeficiency"[All Fields] **AND** "syndrome"[All Fields]) **OR** "acquired immunodeficiency syndrome"[All Fields]) **OR** ("neoplasms"[MeSH Terms] **OR** "neoplasms"[All Fields] **OR** "cancer"[All Fields]) **OR** ("diabetes mellitus, type 2"[MeSH Terms] **OR** "type 2 diabetes mellitus"[All Fields] **OR** "type 2 diabetes"[All Fields]) **OR** ("transplants"[MeSH Terms] **OR** "transplants"[All Fields] **OR** "transplant"[All Fields] **OR** "transplantation"[MeSH Terms] **OR** "transplantation"[All Fields]) **OR** ("autoimmunity"[MeSH Terms] **OR** "autoimmunity"[All Fields]) **OR** ("arthritis"[MeSH Terms] **OR** "arthritis"[All Fields]) **OR** IBD[All Fields] **OR** ("inflammatory bowel diseases"[MeSH Terms] **OR** ("inflammatory"[All Fields] **AND** "bowel"[All Fields] **AND** "diseases"[All Fields]) **OR** "inflammatory bowel diseases"[All Fields] **OR** ("inflammatory"[All Fields] **AND** "bowel"[All Fields] **AND** "disease"[All Fields]) **OR** "inflammatory bowel disease"[All Fields]))	
December 22, 2022	(("epidemiology"[Subheading] **OR** "epidemiology"[All Fields] **OR** "incidence"[All Fields] **OR** "incidence"[MeSH Terms]) **OR** ("epidemiology"[Subheading] **OR** "epidemiology"[All Fields] **OR** "epidemiology"[MeSH Terms]) **OR** ("epidemiology"[Subheading] **OR** "epidemiology"[All Fields] **OR** "prevalence"[All Fields] **OR** "prevalence"[MeSH Terms]) **OR** ("epidemiology"[Subheading] **OR** "epidemiology"[All Fields] **OR** "frequency"[All Fields] **OR** "epidemiology"[MeSH Terms] **OR** "frequency"[All Fields])) **AND** (("diagnosis"[Subheading] **OR** "diagnosis"[All Fields] **OR** "diagnosis"[MeSH Terms]) **OR** detection[All Fields]) **AND** ("legionella"[MeSH Terms] **OR** "legionella"[All Fields]) **AND** (("pneumonia"[MeSH Terms] **OR** "pneumonia"[All Fields]) **OR** (("lung"[MeSH Terms] **OR** "lung"[All Fields]) **AND** ("infections"[MeSH Terms] **OR**	PubMed Central

	"infections"[All Fields] **OR** "infection"[All Fields])) **OR** (("residence characteristics"[MeSH Terms] **OR** ("residence"[All Fields] **AND** "characteristics"[All Fields]) **OR** "residence characteristics"[All Fields] **OR** "community"[All Fields]) **AND** acquired[All Fields] **AND** ("pneumonia"[MeSH Terms] **OR** "pneumonia"[All Fields])) **OR** ("coinfection"[MeSH Terms] **OR** "coinfection"[All Fields]) **OR** (("lung"[MeSH Terms] **OR** "lung"[All Fields]) **AND** abnormality[All Fields]) **OR** (("lung"[MeSH Terms] **OR** "lung"[All Fields]) **AND** abnormality[All Fields]) **OR** (("lung"[MeSH Terms] **OR** "lung"[All Fields]) **AND** ("abnormalities"[Subheading] **OR** "abnormalities"[All Fields] **OR** "congenital abnormalities"[MeSH Terms] **OR** ("congenital"[All Fields] **AND** "abnormalities"[All Fields]) **OR** "congenital abnormalities"[All Fields])))	
January 5, 2023	((incidence) **OR** (distribution) **OR** (epidemiology) **OR** (prevalence) **OR** (frequency) **OR** (specificity) **OR** (sensitivity) **OR** (etiology)) **AND** ((diagnosis) **OR** (detection)) **AND** (legionella) **AND** ((pneumonia) **OR** (lung infection) **OR** (community acquired pneumonia) **OR** (coinfection) **OR** (lung abnormality) **OR** (lung abnormalities))	PubMed
February 10, 2023	((incidence) **OR** (distribution) **OR** (epidemiology) **OR** (prevalence) **OR** (frequency) **OR** (specificity) **OR** (sensitivity) **OR** (etiology)) **AND** ((diagnosis) **OR** (detection)) **AND** (legionella) **AND** ((pneumonia) **OR** (lung infection) **OR** (community acquired pneumonia) **OR** (coinfection) **OR** (lung abnormality) **OR** (lung abnormalities) **OR** (Legionnaires disease) **OR** (Legionellosis) **OR** (Pontiac fever) **OR** (pneumonitis) **OR** (bronchopneumonia))	PubMed
February 10, 2023	Papers outlined in LegionellaDB that either listed identification of a non-pneumophila serogroup 1, Legionella spp., or Legionella pneumophila. Did not include Unknown serogroups/species	LegionellaDB

Supplementary Materials S1. Review protocol

Review title and timescale

1. **Review title:** Epidemiology of non-pneumophila serogroup 1 legionella in people with a diagnosis of pneumonia: A scoping review

2. **Anticipated or actual start date:**
   November 2023
3. **Anticipated completion date:**

February/March 2024

4. Stage of review at time of filling this form out:

Title/abstract screening

Review team details

5. Named Contact:

Zulma Rueda

6. Name Contact Email:

Zulma.rueda@umanitoba.ca

7. Organizational Affiliation of the Review:

[1]Department of Medical Microbiology and Infectious Diseases, University of Manitoba, 745 Bannatyne Ave, Winnipeg, Manitoba, R3E 0J9, Canada

[2]Department of Internal Medicine, University of Manitoba, 750 Bannatyne Ave, Winnipeg, Manitoba, Canada

[3]Shared Health, Diagnostic Services. 1502 – 155 Carlton St, Winnipeg, Manitoba, R3C 3H8, Canada

[4]Cadham Provincial Laboratory, Shared Health, 750 William Ave, Winnipeg, Manitoba, R3E 3J7, Canada

[5]Department of Community Health Sciences, University of Manitoba, 750 Bannatyne Ave, Winnipeg, Manitoba, R3E 0J9, Canada

[6]Universidad Pontificia Bolivariana, Circular 1ª 70-01, barrio Laureles, Medellín, Antioquia, Colombia

8. Review Team Members and Their Organizational Affiliations:

Ryan Ha[1], Ashley Heilmann[1], Sylvain A. Lother[2], Christine Turenne[3], David Alexander[1,4], Yoav Keynan[1,2,5*], Zulma Vanessa Rueda[1,6*]

9. Funding Sources/Sponsors:

Canadian Institute of Health Research

Canadian Research Chair

10. Conflicts of Interest:

N/A

Review methods

11. Review Question(s):

1. Does nucleic acid testing (NAT) increase the detection of non-*pneumophila* serogroup-1 *Legionella* compared to non-NAT?
2. Does immunocompromisation increase the frequency of pneumonia caused by non-*pneumophila* serogroup 1 *Legionella* compared to non-immunocompromised individuals with LD?

12. Literature Search:

Literature searches of online databases PubMed, MEDLine, and LegionellaDB were conducted by a team member. Search strategy reviewed by other members of the team.

13. URL to Search Strategy:

N/A

14. Condition or domain being studied:

Etiologies and diagnosis of Legionnaires' Disease

15. Participants/Population:

Legionnaires' Disease patients regardless of sex or age. Studies involving individuals who are immunocompromised will undergo additional analyses

16. Intervention(s)/Exposure(s):

Genotype-based techniques such as PCR, sequencing

17. Comparator(s)/Control(s):

Phenotype-based techniques such as culture, serology, DFA, and UAT

18. Type of Study to be Included Initially

Original articles, no case studies

19. Primary Outcome:

The results of interest to us are the ability of diagnostic techniques to detect non-*pneumophila* serogroup 1 *Legionella* (stratified by species/serogroups).

20. Data Extraction (selection and coding):

Data will be extracted from retrieved papers to compile and for further analysis. We will collect the following information:

- Reference
- Year of study
- Country(ies) where the study was conducted
- Population of interest
- Sample size disaggregated by sex (female and male)
- Sample type used for Legionella diagnosis
- Technique. If a molecular test was used, which type, which molecular target
- Brand of test/media type
- Outcome
- Any immunosuppressive condition- transplant, HIV (CD4 count), corticosteroids, TNF blockers, smoking, other
- Comorbidities- lung disease, COPD, etc.
- Time of follow-up for those studies that aimed to determine the incidence
- Limitations

21. Risk of bias (Quality) Assessment:

Biases posed by each article will be further analyzed and recognized, in terms of exposure, outcome, and study design.

22. Strategy for Data Synthesis:

The data will be reported as follow: 1) PRISMA flowchart, 2) qualitative synthesis of the full-text articles that were read. 3) A summary table/figure that describes the frequency of non-pneumophila serogroup 1 legionella by country, year of study, population and technique used for diagnosis. 4) A table with the frequency of non-pneumophila serogroup 1 legionella by immunosuppression conditions compared to non-immunosuppressed individuals.

Review General Information

23. Type of review:

Scoping review

24. Language:

English

25. Country:

Canada

26. Reference and/or URL for Published Protocol:

TBD

27. Dissemination Plans:

The finalized paper will be submitted to a peer-reviewed journal.

28. Search terms:

((incidence) OR (distribution) OR (epidemiology) OR (prevalence) OR (frequency) OR (specificity) OR (sensitivity) OR (etiology)) AND ((diagnosis) OR (detection)) AND (legionella) AND ((pneumonia) OR (lung infection) OR (community acquired pneumonia) OR (coinfection) OR (lung abnormality) OR (lung abnormalities) OR (Legionnaires disease) OR (Legionellosis) OR (Pontiac fever) OR (pneumonitis) OR (bronchopneumonia))

More detailed search queries can be found in "Review search log.xlsx" or the supplemental information.

29. Details of any Existing Review of the Same Topic by the Same Authors:

N/A

30. Current Review Status:

Not currently under review

31. Any Additional Information:

N/A

32. Details of Final Report/Publications:

N/A

33. Inclusion Criteria

1) Original research that reports data about the PICOT questions that can be used to calculate incidence, frequency, or prevalence of Legionella with a species or serogroup analysis
2) comparative quantitation using multiple detection methods
3) use of at least one NAT- and one non-NAT-based technique for diagnosis
4) at least 5 cases of LD in the patient group

34. Exclusion Criteria

1) case reports or series of <5 patients
2) missing serogroup or species analysis
3) studies in which patients were only infected with L. pneumophila serogroup 1
4) articles not available in English
5) no abstract
6) examining environmental distribution
7) ongoing trials
8) not done on humans or using human samples
9) non-pneumonic Legionellosis
10) diagnostic techniques are not reported

PICOT for Q1:

Population	*Intervention/exposure (hypothesis)*	*Comparator*	*Outcome*	*Time frame*

Individuals with pneumonia	*Genotype-based techniques such as PCR, sequencing*	*Phenotype-based techniques such as culture, serology, direct fluorescent antibodies, and UAT*	*Incidence, prevalence, and frequency of Legionella compatibility (specific species/strains as stratified by molecular techniques)*	*N/A*

PICOT for Q2:

Population	***Intervention/exposure (hypothesis)***	***Comparator***	***Outcome***	***Time frame***
Individuals with pneumonia who are Immunocompromised	*Genotype-based techniques such as PCR, sequencing*	*Phenotype-based techniques such as culture, serology, direct fluorescent antibodies, and UAT*	*Incidence, prevalence, and frequency of Legionella compatibility (specific species/strains as stratified by molecular techniques)*	*N/A*
